# Interactions of Aqueous Imidazolium-Based Ionic Liquid Mixtures with Solid-Supported Phospholipid Vesicles

**DOI:** 10.1371/journal.pone.0163518

**Published:** 2016-09-29

**Authors:** Patricia Losada-Pérez, Mehran Khorshid, Frank Uwe Renner

**Affiliations:** 1 Institute for Materials Research IMO, Hasselt University, Diepenbeek, Belgium; 2 IMEC vzw, Associated lab IMOMEC, Diepenbeek, Belgium; Universitat Zurich, SWITZERLAND

## Abstract

Despite the environmentally friendly reputation of ionic liquids (ILs), their safety has been recently questioned given their potential as cytotoxic agents. The fundamental mechanisms underlying the interactions between ILs and cells are less studied and by far not completely understood. Biomimetic films are here important biophysical model systems to elucidate fundamental aspects and mechanisms relevant for a large range of biological interaction ranging from signaling to drug reception or toxicity. Here we use dissipative quartz crystal microbalance QCM-D to examine the effect of aqueous imidazolium-based ionic liquid mixtures on solid-supported biomimetic membranes. Specifically, we assess in real time the effect of the cation chain length and the anion nature on a supported vesicle layer of the model phospholipid DMPC. Results indicate that interactions are mainly driven by the hydrophobic components of the IL, which significantly distort the layer and promote vesicle rupture. Our analyses evidence the gradual decrease of the main phase transition temperature upon increasing IL concentration, reflecting increased disorder by weakening of lipid chain interactions. The degree of rupture is significant for ILs with long hydrophobic cation chains and large hydrophobic anions whose behavior is reminiscent of that of antimicrobial peptides.

## Introduction

Ionic liquids (ILs) are a large class of ionic compounds usually composed of an organic cation and an organic or inorganic anion. They present unique physical and chemical properties such as an extremely low vapor pressure, high ionic conductivity, very good chemical and thermal stability, and a broad liquid temperature range (their main phase transition temperature falls below 100°C) [1–3]. Ionic liquids are often referred to as “Green solvents” due to their negligible vapor pressure, which minimizes their release into the atmosphere and renders them non-flammable. All these characteristics provide ILs with great potential for applications in a variety of fields, such as lubricants, electrolytes or bioprocessing, and make them eventually more environmental-friendly and safer substitutes to the traditional organic solvents in many chemical industry processes [4–8]. As a matter of fact, their reputed character as environmentally friendly solvents has motivated a vast number of studies over the last decade [1–8]. Yet, their low volatility renders them odorless and thus hard to detect in the event of a leak to aquatic ecosystems. Their stability and related low biodegradability make their environmental fate a potential problem [9]. In this respect, research on IL-induced toxicity is very scarce compared to that carried out for the physicochemical characterization and their use as solvents in many industries. The toxicity has been studied to date for cell cultures [10, 11] and microorganisms [12]. The fundamental mechanisms behind IL interactions with biological systems and their related toxicity have been rarely studied and are not well understood also, in part, due to the complexity of the systems studied so far. Biomimetic films are important biophysical model systems to elucidate fundamental aspects and mechanisms relevant for a large range of biological interaction ranging from biosignaling to drug reception or toxicity.

Among the different kinds of biomolecules, the interaction of ILs with lipid-based structures is of considerable relevance, since lipids and in particular phospholipids constitute the building blocks of the cell membrane, i.e. the protective barrier separating the contact of ILs with a living cell. Fundamental research has focused on MD simulations [13–15] and on few experimental studies for model lipid systems, such as large unilamellar vesicles (LUVs) using mainly luminescence, differential scanning calorimetry and neutron reflectometry [16–20]. The study of ILs interactions with solid-supported lipid layers is mainly limited to the stability of 2D-supported lipid bilayers (SLBs) exposed to very high concentrated aqueous mixtures of ILs (much higher than their CAC) by using atomic force microscopy and quartz crystal microbalance [21, 22]. The study of the effect of diluted aqueous mixtures of ILs on solid-supported membranes is so far restricted to phosphonium based ILs [23]. Further studies of different supported layer geometries and systems are thus necessary to get a deeper understanding in a wider concentration range. Being 3D-supported systems, supported lipid vesicles (SLV) layers offer specific advantages and represent a geometry in between extended flat layers and real cells. SLV are thus interesting layers for both fundamental biophysical studies and pharmaceutical screening applications [24–28].

In this work, we have used quartz crystal microbalance with dissipation monitoring (QCM-D) to examine the effect of imidazolium-based ILs on SLVs varying two parameters: the length of the cation and the nature of the anion in the low concentration regime (below the CAC for most of the studied systems). Advantages of QCM-D are its compact set-up with a small quantity of sample solution needed, a low temperature equilibration time, and no need for labelling molecules. Traditionally, QCM-D has been mainly used to characterize vesicle adsorption kinetics and formation of SLBs and bilayer-protein interactions, see, for instance [29–32]. Only recently, it was proposed as a method to study structural layer changes upon the main phase transition of phospholipid layers [33–36]. Apart from looking at the stability of the supported vesicle layer upon exposure to the aqueous IL mixture, we will make use of a recently developed strategy to examine the resulting changes in lipid phase behaviour [37]. Specifically, we calculate the temperature derivative of the directly measured frequency shift response on layers of lipid + IL and observe extrema which stand as a token of the lipid main phase transition. We have chosen a saturated phospholipid, dimyristoilphosphatidylcholine (DMPC), and ILs with imidazolium-based cations with hydrophilic and hydrophobic anions. In addition the effective shear viscosity temperature profile have been obtained by means of a Voigt-based viscoelastic model to analyze its changes upon the main phase transition. Both approaches yield consistent information on the IL-induced changes on the phase transition and viscoelastic properties of the lipid layers under study. Our results can be explained as a function of the cation length and the degree of hydrophobicity of the anion.

## Materials and Methods

**Fig 1:**
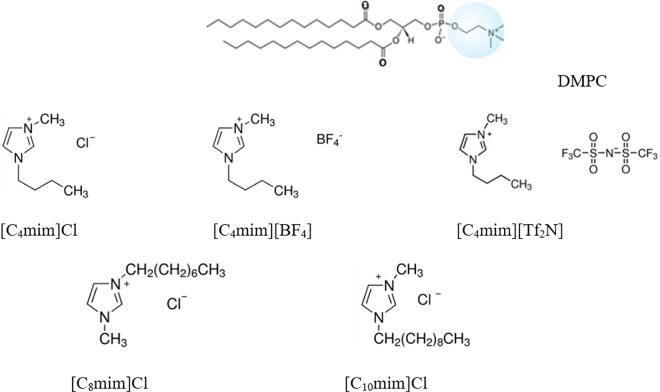
Schematic chemical structure of the lipid and ionic liquids studied in this work.

DMPC was purchased from Avanti Polar Lipids (Alabaster, AL). 1-butyl-3-methylimidazolium chloride [C_4_mim]Cl, assay ≥ 98%, 1-octyl-3-methylimidazolium chloride [C_8_mim]Cl, assay ≥ 97%, 1-decyl-3-methylimidazolium chloride [C_10_mim]Cl, assay 96%, 1-Butyl-3-methylimidazolium tetrafluoroborate [C_4_mim][BF_4_], assay ≥ 97%, 1-Butyl-3-methylimidazolium bis(trifluoromethylsulfonyl)imide [C_4_mim][Tf_2_N], assay ≥98%, were purchased from Sigma-Aldrich (Diegem, Belgium). A schematic of the chemicals used is given in Fig 1. A list including the CACs of the ionic liquids used can be found in S1 Table). Spectroscopic grade chloroform assay 99.3% (stabilized with about 0.6% ethanol) was obtained from Analar (Normapur). HEPES buffer (pH 7.4) consisting of 10 mM HEPES from Fisher Scientific (assay 99%) and 150 mM NaCl-from Sigma-Aldrich (assay ≥ 99.5%) was used for hydration of the dried lipids. The quantities of lipids to reach the desired mixture concentrations were determined gravimetrically using a Sartorius balance yielding a maximal mole fraction uncertainty of ± 0.002.

### Vesicle preparation

The lipid powder was first dissolved in spectroscopic grade chloroform and the solvent was then evaporated under a mild flow of nitrogen in a round bottomed flask. The resulting lipid film was kept under vacuum overnight to remove residual solvent. Then, the lipid was hydrated with HEPES buffer. Hydration to 0.5 mg/ml was carried out under continuous stirring in a temperature-controlled water bath at 45°C, well above the main phase transition temperature of DMPC (*T*_m_ ~ 24°C). Small unilamellar vesicles (SUVs) were formed by extrusion through a filter support (Avanti Polar Lipids) with a pore size of 100 nm for 25 times. Vesicle effective sizes and polydispersities were determined by dynamic light scattering (Zeta Pals, Brookhaven Instruments Corporation). The vesicle dispersions were stored at 4°C and used within 2 days.

### Quartz crystal microbalance with dissipation

Quartz crystal microbalance with dissipation monitoring (QCM-D) is an acoustic surface-sensitive technique based on the inverse piezoelectric effect. The application of an AC voltage over the sensor electrodes causes the piezoelectric quartz crystal to oscillate at its acoustic resonance frequency. As a result, a transverse acoustic wave propagates across the crystal, reflecting back into the crystal at the surface. When the AC voltage is turned off, the oscillation amplitude decays exponentially, this decay is recorded and the frequency (*f*) and the energy dissipation factor (*D*) of different overtones are extracted [24]. The dissipation *D* is the ratio between the dissipated energy during one vibration cycle and the total kinetic and potential energy of the crystal at that moment and can reveal insight in the viscoelastic behavior of surface films.

QCM-D uses a compact sample and crystal compartment attached to a micro-fluidic system. When molecules adsorb to an oscillating quartz crystal, water (or buffer) couples to the adsorbed material as an additional dynamic mass via direct hydration and/or entrapment within the adsorbed film. The layer is sensed as a viscoelastic hydrogel composed of the molecules and the coupled water together. The adsorbed layer is described by a frequency-dependent complex shear modulus, defined as [38,39]:

$$G={G\prime +iG\prime \prime =\mu }_{f}+2{\pi if\eta }_{f}=\mu_{f}\left(1+2\pi i\chi \right),$$ (1)

where *G’* and *G*” stand for energy storage and dissipation, respectively, *f* is the oscillation frequency, *μ*_f_ is the elastic shear storage modulus, *η*_f_ is the shear viscosity, and χ = *η*_f_ /*μ*_f_, is the relaxation time of the layer.

For the current measurements, we have used QCM-D on a Q-sense E4 instrument (Gothenborg, Sweden) monitoring the frequency shift Δ*f* and the dissipation change Δ*D*. Q-sense E4 also enables heating or cooling temperature scans from 15°C to 50°C. AT-cut quartz crystals with Au coating (diameter 14 mm, thickness 0.3 mm, surface roughness 3 nm and resonant frequency 4.95 MHz) were used. The Au-coated quartz sensors were cleaned with a 5:1:1 mixture of Milli-Q water (conductivity of 0.055 S cm^–1^ at 25°C), ammonia and hydrogen peroxide, and were UV-ozone treated with a Digital PSD series UV-ozone system from Novascan for 15 min, followed by rinsing in milli-Q water and drying with N_2_. The changes in Δ*f*/n and in Δ*D* were monitored at five different overtones (from 3^rd^ to 11^th^, the fundamental frequency is rather unstable reaching the farthest out to the edge of the sensor and likely affected by the O-ring). The temperature stability at constant temperature was ± 0.02°C. For the phase behavior study, temperature scans with alternating heating and cooling were performed at a rate of 0.4°C/min, maintaining 60 minutes of stabilization between successive ramps. For each sample, experiments were carried out twice in independent runs in order to test the repeatability of the measurements.

## Results and Discussion

### Stability of DMPC vesicle layers exposed to imidazolium-based ionic liquids

**Fig 2:**
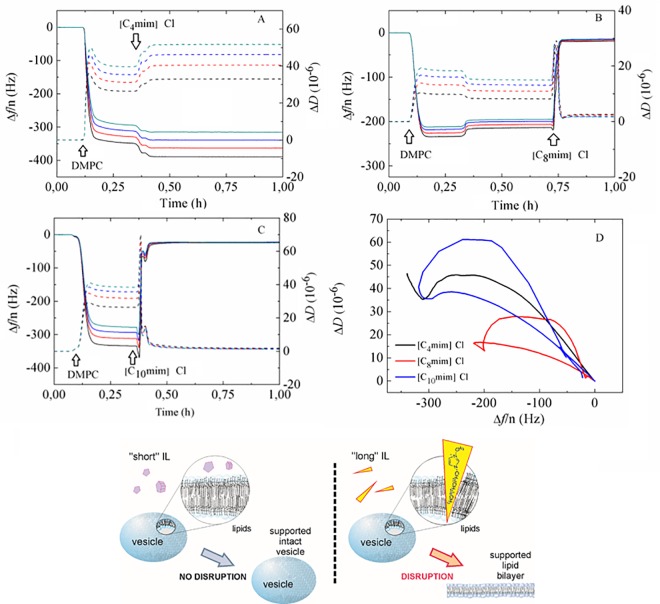
Time evolution of Δ *f*/n (solid lines) and Δ*D* (dashed lines) during a QCM-D experiment of DMPC vesicle adsorption exposed to (A) [C_4_mim]Cl, (B) [C_8_mim]Cl and (C) [C_10_mim]Cl at a concentration of 50 mM. (D) Δ*D vs* Δ*f*/n plot for all three mixtures. Black arrows indicate the time when a given sample was added. (E) Schematic representation of the action of short chain and long chain ionic liquids on DMPC SLVs.

The experiments were carried out at a temperature of 30°C using commercial gold-coated QCM-D substrates. This system is known to favor intact vesicle adsorption and results in a stable SLV [29,40]. Here, the effect of aqueous IL mixtures on a DMPC SLV has been studied for systems with first, common anion Cl^-^ and increasing cation length and second, systems with common cations [C_4_mim] and different anion nature. After obtaining a stable baseline in the QCM-D measurement in HEPES buffer, the 0.5 mg/mL DMPC vesicle dispersions were introduced at a rate of 50 μL/min for 20 minutes until a clear signature of a vesicle layer formation was observed. The large frequency shift Δ*f* and dissipation Δ*D* values observed and the fact that the different overtones do not overlap denotes the deposition of an acoustically non-rigid vesicle layer. Then, the IL aqueous mixture (mostly) with a fixed concentration of 50 mM was injected for 15 minutes and the pump was stopped. For [C_4_mim]Cl, [C_4_mim][BF_4_] and [C_8_mim]Cl this concentration lies clearly below their respective CACs [41, 42]. At a 50 mM concentration of [C_10_mim]Cl- the presence of aggregates cannot be ruled out judging from the literature concentration interval where the CAC takes place (45–60 mM) [43]. For [C_4_mim][Tf_2_N], its solubility limit is 20 mM. For concentrations larger than 20 mM no visible precipitation was observed before introducing the ionic liquid in the measuring cell [44, 45]. Fig 2 illustrates the effect of common anion systems with [C_N_mim] cation lengths N = 4, 8, and 10. As it can be observed, the interaction of [C_4_mim]Cl with the DMPC vesicle layer is reflected only in a slight decrease of Δ*f* and increase of Δ*D* in Fig 2A, indicating a small, partial insertion of the ionic liquid molecules into the adsorbed vesicle layer. Though not shown, further rinsing with HEPES buffer resulted in none or only a very slight decrease of Δ*f* and increase of Δ*D*, leaving the layer practically unaltered (see S1 Fig). Upon addition of long-chain length IL molecules (C_N_mim with N = 8, 10, shown in Fig 2B and 2C), Δ*f* decreases first, with the corresponding increase in Δ*D* indicating initial adsorption of the IL mixture. Immediately following is then a significant rapid increase of Δ*f* and decrease of Δ*D* reflecting complete vesicle rupture (with trapped buffer release), until stable and overlapping overtone values Δ*f ~* –23 Hz, Δ*D* ~ 0 are reached. These final values correspond to the presence of a thin, rigid supported lipid bilayer [29], and the frequency and dissipation signatures upon addition of the ionic liquid mixture are reminiscent of the so-called disrupting carpet mechanism of antimicrobial forming peptides [32, 46]. Such behavior reflects the potential of ILs as alternative disrupting agents for antimicrobial peptides or antibiotics. This behaviour was recently observed for long chain phosphonium-based ionic liquids onto anionic biomimetic membranes [23]. Further rinsing with HEPES buffer removed minor residues of lipid + IL complexes from the sensor surfaces (see panels B and C of S1 Fig). An additional Δ*D* − Δ*f* plot shown in Fig 2D, eliminates time as an explicit adsorption parameter and illustrates the different patterns observed upon addition of short and long chain cation with a common Cl anion ILs. Specifically, both SLVs with [C_8_mim]Cl and [C_10_mim]Cl show a so-called re-entrant pattern, characteristic for vesicle layer disruption and bilayer formation, while the SLV exposed to [C_4_mim]Cl shows intact vesicle layer formation and subsequent interaction with the IL without significant disruption. These results can be further rationalized as follows: given Cl is a hydrophilic anion, all three ILs exhibit amphiphilic interactions driven by the cation and they tend to spontaneously insert into the lipid bilayer. As the cations insert, the imidazolium ring and the alkyl chain are strongly associated with the lipid head and tail groups, respectively. The long hydrocarbon sequence of long chain cations displays stronger hydrophobic interactions with the lipid tail group thus promoting deep and irreversible insertion and disruption as is apparent from our experiments. In turn, short chain [C_4_mim]Cl molecules only slightly incorporate into the vesicle bilayer wall (they might eventually detach) driven by weaker hydrophobic interactions than their long chain counterparts. These two kinds of IL action are schematically summarized in Fig 2E. We thus find that the interaction of the ionic liquid with lipid bilayers can be controlled by the ratio of the hydrophilic and the lipophilic parts of the IL ions.

**Fig 3:**
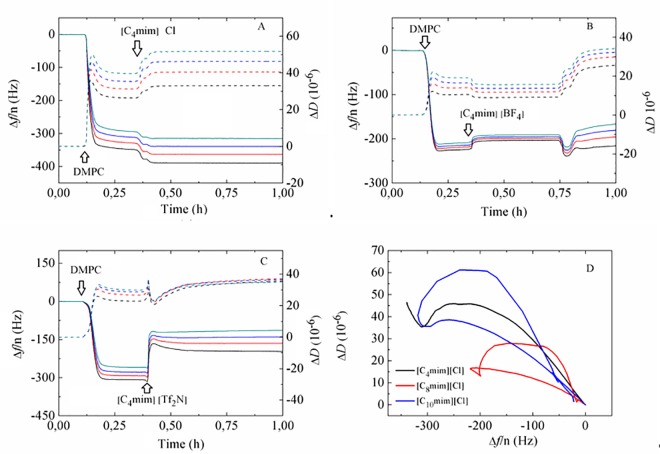
Time evolution of Δ*f*/n (solid lines) and Δ*D* (dashed lines) during a QCM-D experiment of DMPC vesicle adsorption exposed to (A) [C_4_mim]Cl, (B) [C_4_mim][BF_4_] and (C) [C_4_mim][Tf_2_N] at a concentration of 50 mM. (D) Δ*D vs* Δ*f*/n plot for all three mixtures. Black arrows indicate the time when a given sample was added.

Fig 3 shows the Δ *f* and Δ *D* responses upon addition of systems with a common [C_4_mim] cation and anions of different nature, namely Cl, [BF_4_] and [Tf_2_N]. The hydrophilic Cl ion shows no tendency to disrupt the vesicle layer (see Fig 3A), remaining either in solution hydrated in the aqueous phase or near the charged groups of the bilayer [18]. Regarding the more hydrophobic [Tf_2_N] anion, a moderate tendency to insert into the bilayer and partial disruption of the SLV can be observed in Fig 3C, where upon [C_4_mim][Tf_2_N] addition, the apparent mass of the film (Δ*f*/n) decreases significantly. According to a report in the literature a phase separation of [C_4_mim][Tf_2_N] in equilibrium with ions in remaining solution can occur at concentrations above the solubility limit, limiting the penetration of ions into the bilayer and thus inhibiting complete disruption [47]. In turn, the [BF_4_] anion is less hydrophobic than [Tf_2_N] [48] and displays an intermediate behavior between Cl and [Tf_2_N]. The Δ*D* − Δ*f* plot shown in Fig 3D illustrates the different patterns observed upon addition of ILs of different anion with a common cation. Specifically, the SLV exposed to [C_4_mim]Cl shows intact vesicle layer formation and subsequent interaction with the IL without significant disruption. The layers exposed to [C_4_mim][BF_4_] and [C_4_mim][Tf_2_N] show a re-entrant pattern, the former being much smaller than the latter. This suggests very mild layer disruption by [C_4_mim][BF_4_] and partial insertion and disruption by [C_4_mim][Tf_2_N]. Subsequent rinsing with buffer removed lipid-IL complexes or weakly-adsorbed vesicles (see panels D and E in S1 Fig). The above-exposed dependence on cation length and nature of the anion agrees with a scenario proposed by recent MD simulations [13, 14, 47]. At this point, it is worth mentioning that the effect of osmotic stress on vesicle rupture by increasing membrane tension is, although small, difficult to quantify and discriminate in our case, since we are introducing ILs in the same buffer as the one where vesicles were produced. Strong osmotic stress has been used to rupture small unilamellar vesicles by exchanging high ionic strength buffer with deionized water (so-called osmotic shock) [49] and to promote vesicle spreading in giant unilamellar vesicles with aquaporin-0 [50].

### Phase behavior of DMPC incubated with ILs

**Fig 4:**
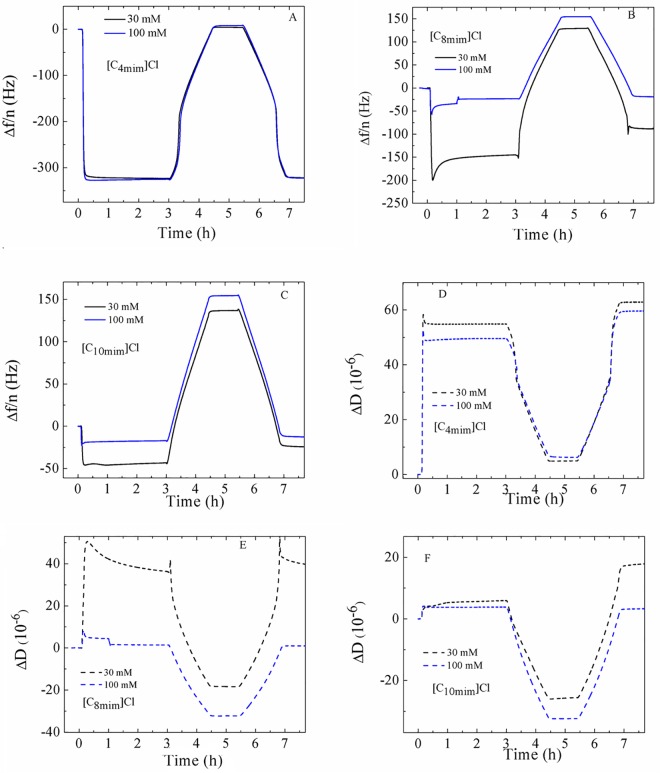
Overview of the time dependence of the Δf/n and ΔD responses of systems containing 30 mM and 100 mM [C_4_mim]Cl, [C_8_mim]Cl and [C_10_mim]Cl onto a gold-coated QCM-D quartz sensor. Results are displayed for overtone 7^th^.

In order to evaluate the influence of the IL on the phase behavior of DMPC SLV, we incubated unilamellar vesicles together with IL aqueous mixtures at different concentrations. The vesicle size distribution in the presence of each IL was measured using dynamic light scattering after an incubation time of at least 20 minutes. The results are shown in Table 1, where the effective diameter, the half width of the size distribution and the polydispersity index are included. Overall, the change in size of the vesicles in the presence of IL is not significant compared to pure DMPC vesicles (sizes ranging between 100−128 nm). For [C_8_mim]Cl, [C_10_mim]Cl and [C_4_mim][Tf_2_N], the size slightly decreases with increasing concentration indicating some vesicle alternation or partial disruption, while no effect is observed for [C_4_mim]Cl-and [C_4_mim][BF_4_]. However, in the presence of ILs the width of the distribution function clearly increases for all but [C_4_mim][Tf_2_N]. For all ILs, the polydispersity index, a measure of size distribution increases with the amount of IL. The presence of IL is thus leading to a decrease in vesicle stability. At the highest concentration for [C_10_mim]Cl at 100 mM, which is above the respective ionic liquid CAC [43] the formation of unstable liposomes was observed. After the size measurements, DMPC + ILs aqueous mixtures were introduced in the QCM-D sensors and the formation of SVLs and subsequent phase transition behavior were monitored. Fig 4 shows an example of the significant differences in adsorption behavior and thermal responses between vesicles containing the same concentration of ILs with short chain and long chain cation with the same Cl anion. Vesicles containing [C_4_mim]Cl display large Δf/n ~ –300 Hz and ΔD ~ 50·10^−6^ plateau values upon adsorption and formation of a stable and viscoelastic vesicle layer. Vesicles incubated with [C_8_mim]Cl exhibit smaller Δf/n and ΔD values than th ones containing the same concentration of [C_4_mim]Cl. For the 30 mM sample, continuous rupture can be observed before the start of the heating and cooling cycles. Vesicle stability decreased judging from the difference in the plateau values before and after the thermal cycles. The system with 100 mM [C_8_mim]Cl exhibits initial Δf/n ~ –50 Hz and ΔD ~ 6·10^−6^ values followed by gradual vesicle rupture and very small resulting plateau values large Δf/n ~ –30 Hz and ΔD ~ 2·10^−6^, denoting large significant disruption. Vesicles containing [C_10_mim]Cl exhibit very small plateau values and formation of rigid layers, indicating that vesicle rupture has taken place previously. For 100 mM [C_10_mim]Cl, the very small values Δf/n < 20 Hz and ΔD ~ 3·10^−6^ suggest that the adsorbed layer did not consist of intact vesicle layers. An overview of vesicle adsorption and layer formation for the remaining systems can be found in S2 and S3 Figs. Systems containing [C_4_mim][BF_4_] show a similar response to [C_4_mim]Cl, while those containing [C_4_mim][Tf_2_N] display smaller Δf/n than its Cl and [BF4] anion counterparts at the same concentration, indicating that partial disruption has occurred.

**Table 1:** Mean diameter and half-width of the size distribution of DMPC vesicles incubated in the presence of ILs. The number in parentheses is the polydispersity index.

Concentration IL (mM)	Vesicle diameter (nm)				
	[C_4_mim]Cl	[C_8_mim]Cl	[C_10_mim]Cl	[C_4_mim][BF_4_]	[C_4_mim][Tf_2_N]
**0**	121 ± 20 (0.06)	122 ± 30 (0.07)	122 ± 29 (0.05)	123 ± 30 (0.05)	125 ± 25 (0.07)
**5**	123 ± 23 (0.04)	115 ± 30 (0.06)	112 ± 42 (0.08)	123 ± 38 (0.09)	111 ± 30 (0.06)
**15**	124 ± 37 (0.08)	112 ± 35 (0.09)	112 ± 37 (0.09)	124 ± 31 (0.07)	116 ± 29 (0.05)
**30**	122 ± 49 (0.08)	112 ± 32 (0.09)	114 ± 59 (0.14)	127 ± 46 (0.11)	115 ± 27 (0.05)
**100**	123 ± 53 (0.11)	100 ± 39 (0.11)	−	128 ± 48 (0.13)	121 ± 30 (0.08)

These results, as we shall see below, agree with the observed phase behavior by QCM-D temperature scans.

### Viscoelastic analysis of the SLV phase behavior

**Fig 5:**
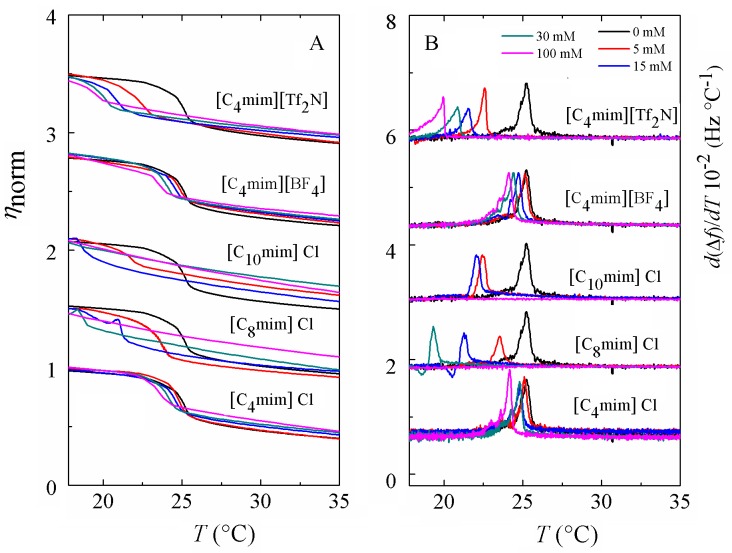
(A) Temperature dependence of the calculated effective viscosity *η*_norm_ normalized as $\eta_{norm}=\frac{\eta (t)}{\eta (t=0)}$ of a supported vesicle layer on a gold-coated QCM-D quartz sensor. (B) Temperature profiles of the first-order temperature derivative of the frequency shifts. For display reasons, the data have been shifted upwards a constant value.

We have used a Kelvin-Voigt based model introduced by Voinova *et al*. assuming that the vesicle layer has uniform thickness, film density, Newtonian bulk fluid and no-slip conditions (perfect coupling onto the quartz sensor). As a result, │ Δ*f* /n│ and Δ*D* can be expressed in terms of the film density *ρ*_*f*_, viscosity *η*_*f*_ and thickness *h*_*f*_ [38, 39]:

$$\Delta f≅\frac{1}{2{\pi \rho }_{q}h_{q}}h_{f}\rho_{f}\omega \left(1+\frac{2h^{2}\chi }{3\delta^{2}\left(1{+\chi }^{2}\right)}\right),$$ (2)

$$\Delta D≅\frac{2t^{3}\rho_{l}f}{3{\pi f}_{0}\rho_{q}h_{q}}\left(\frac{1}{\delta^{2}\left(1{+\chi }^{2}\right)}\right),$$ (3)

$$tan\delta =\frac{1}{\chi }\left(\frac{2\pi f\eta_{l}}{\mu_{l}}\right),$$ (4)

$$\delta =\sqrt{\frac{2\eta_{l}}{\rho_{l}f}},$$ (5)

where *f* is the frequency of a given overtone, *f*_*0*_ is the fundamental frequency, *ω* = 2π*f*, *h* denotes thickness of the quartz and *μ* shear modulus and δ is referred to the penetration depth of a propagating shear wave into the vesicle film. The subscripts q and f refer to quartz and to the vesicle layer, respectively, and χ the ratio between *μ*_*f*_ and *η*_*f*_. The data of several overtones (3^rd^ to 11^th^) were fitted using the software Qtools (Q-Sense AB, Sweden) keeping as fixed parameters the density of the lipid layer 1.06 g·cm^-3^ [51], the density of the fluid 1.0 g·cm^-3^ and the viscosity of the fluid 1 mPa·s. The viscosity values should be taken as effective and not as absolute values because the model assumes a homogeneous vesicle layer and our vesicle dispersions were not perfectly monodisperse. Fig 5A displays the temperature dependence of the normalized effective shear viscosity $\eta_{norm}=\frac{\eta (t)}{\eta (t=0)}$ of all the systems under study upon heating. For the sake of better comparison, we chose to normalize the calculated viscosities since the homogeneity of all layers might be different. Upon cooling, the viscosity responses show a similar shape as upon heating with a slight degree of hysteresis, which is a characteristic feature of first-order transitions [52]. The phase transition from the gel to the liquid disordered state is characterized by a decrease of the shear viscosity from a more viscous state to a less viscous one. At a given temperature a sudden decrease in *η* is observed denoting the starting point of the phase transition. Once all the lipid molecules have completed the phase conversion, a regular behavior is recovered and all lipids are in the liquid-disordered phase. The transition is very cooperative, judging from the small temperature interval where it takes place, as observed in previous works for phase transitions of pure lipids [33, 34, 36]. In mixtures containing ILs a common pattern of behavior is observed with increasing IL concentration: the jump in *η*(*T*) is shifted towards lower temperatures and the viscosity in the fluid phase increases. For short chain cations with Cl or [BF_4_] anion, the shift in the transition temperature is rather small. In turn, the shift is dramatic for ILs with long chain cations or hydrophobic anion. As the IL concentration increases, the transition takes place in a wider temperature range and the jump in *η* changes from a steeper to a shallower slope, indicating the loss of the intermolecular cooperation between phospholipid molecules induced by the incorporation long chain cations and hydrophobic anions. For mixtures with [C_10_mim]Cl no transition is detectable from 30 mM (no jump in *η*), reflecting the complete disruption of the vesicle layer. The increase in viscosity in the fluid phase is more significant for vesicle layers exposed to long chain cations given their stronger van der Waals interactions and layers exposed to larger anions due to the higher molecular mass.

### Frequency derivative analysis of the phase behavior

The signature of a temperature–driven phase transition of a lipid SLV is characterized by an anomalous behavior in the frequency and dissipation shift responses [35, 37]. A useful way of looking at it is to plot the temperature derivative of the frequency *d*(Δ*f*/n)/*dT*, which displays a maximum around temperature where the main phase transition takes place. The advantage of plotting the derivative is that unlike *η*(*T*) curves, the determination of the onset and completion temperatures is more straightforward. The shape of the frequency shift curve for pure DMPC is reminiscent of the enthalpy jump and its heat capacity derivative from calorimetric measurements [34, 36]. Fig 5B shows the results of the thermal analysis of the five kinds of ILs used. In the presence of ILs with short cation length and hydrophilic anion the phase transition temperature is slightly shifted downwards upon increasing the concentration of the IL. In addition, the shape and width of the maximum hardly changes, indicating that [C_4_mim]Cl and [C_4_mim][BF_4_] slightly insert and disorder the SLV. In turn, ILs with long alkyl chains or hydrophobic anion exhibit a dramatic temperature shift in a much larger range upon increasing IL concentration. The peaks are broader indicating the loss of cooperativity upon IL incorporation on the vesicle layer and show peculiar shapes. For all concentrations of [C_8_mim]Cl-and [C_10_mim]Cl-the maximum shifts towards lower temperatures, indicating that the IL significantly weakens the van der Waals interactions between the alkyl chains in DMPC. Vesicle layers exposed to [C_8_mim]Cl, whose CAC is ~ 102 mM [41] display large transition temperature shifts at 15 mM and 30 mM and no transition in the studied temperature range at 100 mM, a concentration very close to its CAC. Likewise, the main transition is suppressed within the studied temperature range at 30 mM of [C_10_mim]Cl, a concentration very close to its CAC ~ 45–60 mM [44]. The ionic liquid with the longest chain [C_10_mim]Cl undergoes the largest deviation in main transition temperature towards lower temperatures and thus the highest capability to perturb the lipid membrane. At high [C_8_mim]Cl-and [C_10_mim]Cl-concentrations, the derivative maximum displays a peculiar shape. Despite its reproducibility, it is not straightforward to interpret the shape of the curve, which corresponds to an inflection point in the Δf/n data and points to some, here unknown, change in internal structure or intermolecular interaction in the layer. The results show a similar trend to differential scanning calorimetry measurements of multilamellar vesicles of a longer phospholipid, DPPC mixed with [C_8_mim][BF_4_] [18]. At an IL concentration of 50 mM the authors observe a shift of 10°C and the heat capacity curves display a peculiar shape. Regarding [C_4_mim][Tf_2_N], a gradual decrease of the phase transition has been observed with increasing concentration of IL. [Tf_2_N] anions follow the [C_4_mim] cation into the bilayer and reside close to the intermediate region between the polar head and the hydrocarbon tails of DMPC [47], thus giving rise to a larger area per molecule and stabilizing the liquid disordered phase.

## Conclusions

The effect of aqueous imidazolium-based ionic liquid mixtures on solid-supported biomimetic membranes of the phospholipid DMPC was studied in a systematic way using quartz crystal microbalance with dissipation QCM-D.

The stability of the supported vesicle layer upon exposure to aqueous IL mixtures at a concentration below the CAC was evaluated. ILs with short chain cation and hydrophilic anion incorporate into the vesicle layer inducing very mild disorder, as inferred from the small changes in frequency and dissipation responses and small shifts in phase transition temperature. In turn, long chain cations penetrate into the SLV and rapidly disrupt and destroy it yielding a rigid supported lipid bilayer, their QCM-D fingerprint being reminiscent of the carpet mechanism of antimicrobial peptides disrupting action. Results indicate that the interactions with phospholipid biomimetic membranes are modulated by the hydrophobic long chain cations or the large hydrophobic anions. Furthermore these conclusions were confirmed by two complementary viscoelastic approaches, the Voigt-based viscoelastic modeling and the temperature-dependence of the (mass sensitive) frequency shifts. The shear viscosity and frequency-derivative temperature profiles display a clear shift downwards of the main phase transition upon the addition of ILs. The effect is more significant upon increasing IL concentration, cation chain length and anion hydrophobicity, which reflects the decreased degree of organization induced by the incorporation and eventual destabilization of the membrane by long chain cations or hydrophobic anions. The disruption of the bilayer structure results in broader transition ranges, i.e. peculiar shapes. Complementary dynamic light scattering measurements indicate that the size of the vesicles does not show significant changes in the presence of ILs as compared to pure lipid vesicles. At concentrations above the CAC for the IL with the longest chain cation, unstable dispersions were observed.

This work thus evidences the importance of hydrophobic interactions between ILs and biomimetic membranes even at low IL concentrations, and reflects the potential of ILs as alternative disrupting agents for antimicrobial peptides or antibiotics. In addition, it serves as a reference for further studies on more complex vesicles containing negatively charged and unsaturated lipids.

## Supporting Information

S1 Fig Time evolution of Δ*f*/n (solid lines) and Δ*D* (dashed lines) during a QCM-D experiment of DMPC vesicle adsorption exposed to (A) [C_4_mim]Cl, (B) [C_8_mim]Cl, (C) [C_10_mim]Cl, (D) [C_4_mim][BF_4_] and (E) [C_4_mim][Tf_2_N] at a concentration of 50 mM.
(DOCX)

S2 Fig Time dependence of the Δf/n and ΔD responses of systems containing 30 mM and 100 mM [C_4_mim][BF_4_] onto a gold-coated QCM-D quartz sensor. Results are displayed for overtone 7^th^.
(DOCX)

S3 Fig Time dependence of the Δf/n and ΔD responses of systems containing 30 mM and 100 mM [C_4_mim][Tf_2_N] onto a gold-coated QCM-D quartz sensor. Results are displayed for overtone 7^th^.
(DOCX)

S1 Table Literature values of the CACs of the studied ionic liquid aqueous mixtures. (DOCX)
