# Supplementary figures and images for: Interactions of Aqueous Imidazolium-Based Ionic Liquid Mixtures with Solid-Supported Phospholipid Vesicles
Source: PLoS One. 2016 Sep 29;11(9):e0163518. doi: 10.1371/journal.pone.0163518 (PMC5042501; doi:10.1371/journal.pone.0163518)

**S1 Fig**


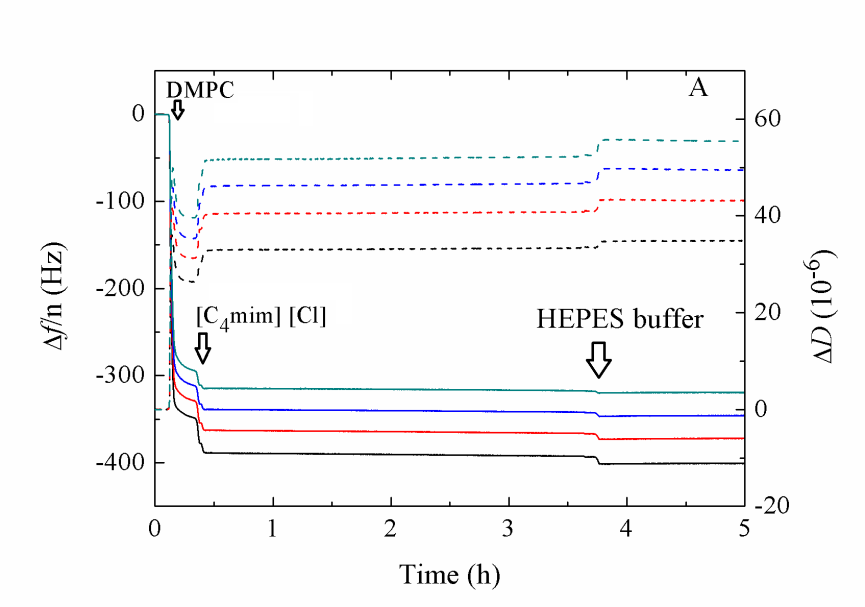

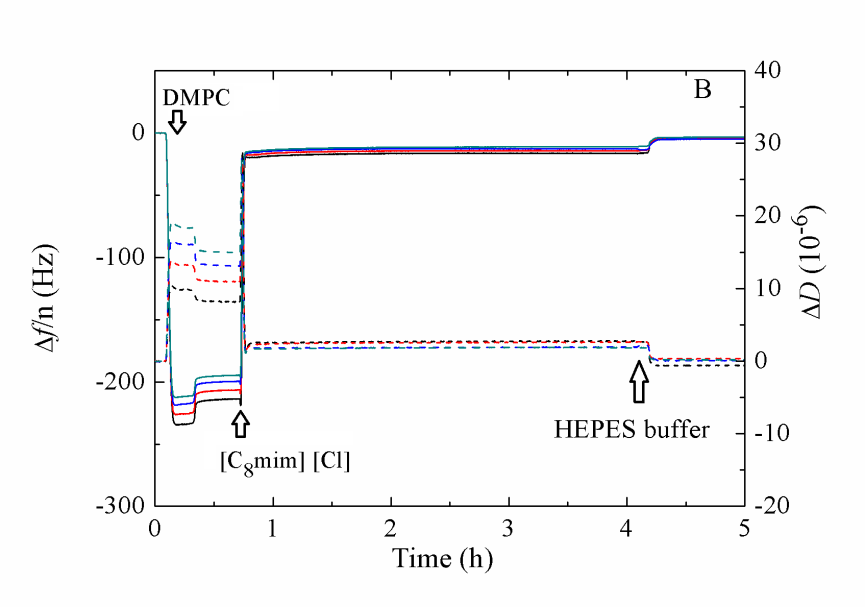


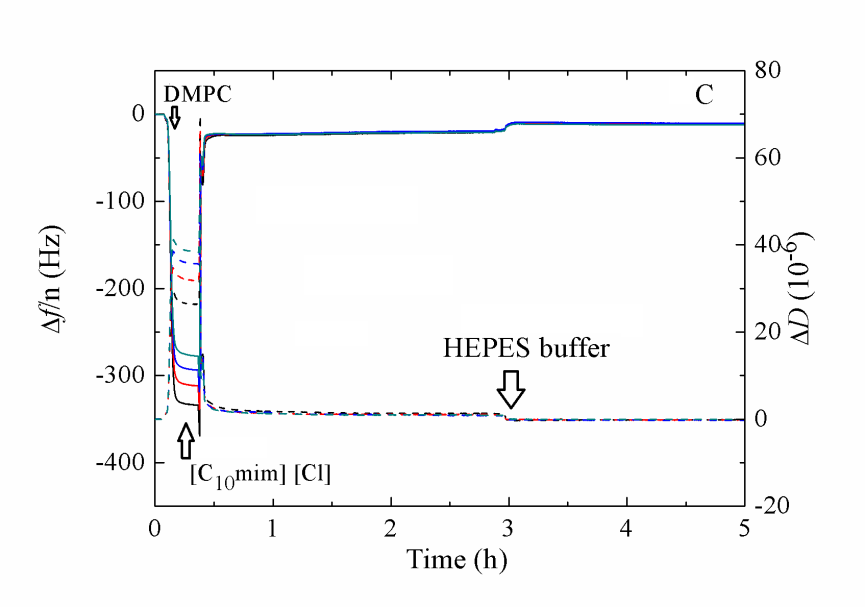

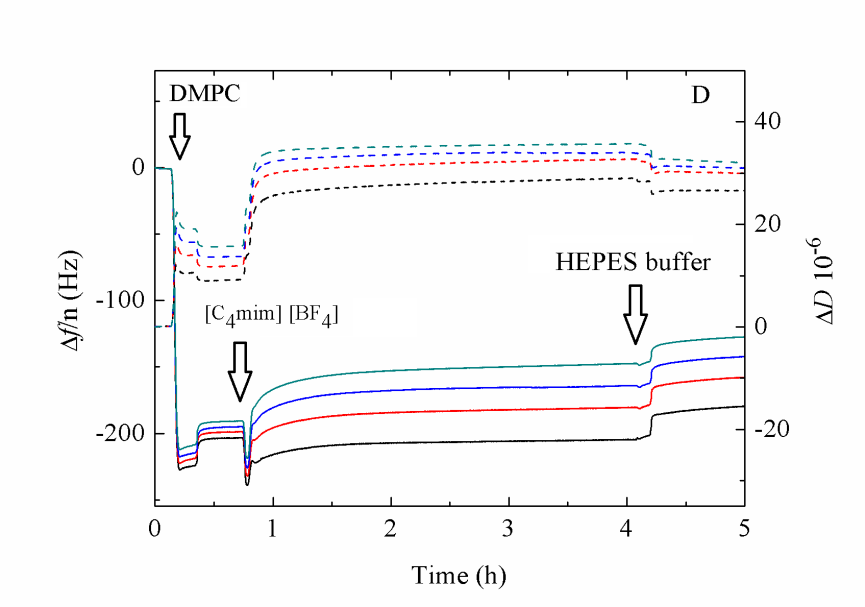


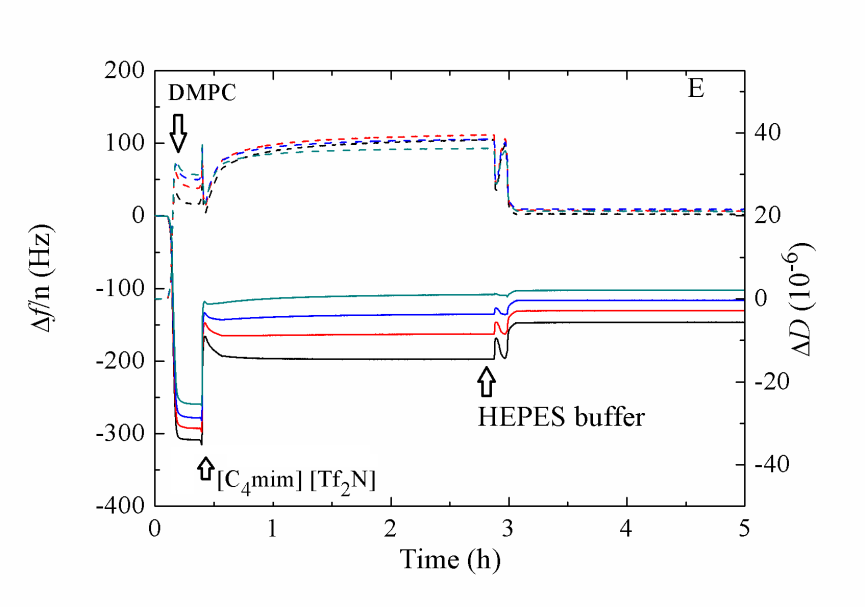

Supplement: S1 Fig — Time evolution of Δf/n (solid lines) and ΔD (dashed lines) during a QCM-D experiment of DMPC vesicle adsorption exposed to (A) [C4mim]Cl, (B) [C8mim]Cl, (C) [C10mim]Cl, (D) [C4mim][BF4] and (E) [C4mim][Tf2N] at a concentration of 50 mM. (DOCX) [file pone.0163518.s001.docx]
