# Supplementary material for: Interactions of Aqueous Imidazolium-Based Ionic Liquid Mixtures with Solid-Supported Phospholipid Vesicles
Source: PLoS One. 2016 Sep 29;11(9):e0163518. doi: 10.1371/journal.pone.0163518 (PMC5042501; doi:10.1371/journal.pone.0163518)
Supplement: S2 Fig — Results are displayed for overtone 7th. (DOCX) [file pone.0163518.s002.docx]

S2 Fig
