# Supplementary material for: Interactions of Aqueous Imidazolium-Based Ionic Liquid Mixtures with Solid-Supported Phospholipid Vesicles
Source: PLoS One. 2016 Sep 29;11(9):e0163518. doi: 10.1371/journal.pone.0163518 (PMC5042501; doi:10.1371/journal.pone.0163518)
Supplement: S1 Table — (DOCX) [file pone.0163518.s004.docx]

S1 Table

| **System** | **CAC (mM)** | **Reference** |
| --- | --- | --- |
| **[C_4_mim]Cl** | 1000 | 41 |
| **[C_8_mim]Cl** | 102 | 41 |
| **[C_10_mim]Cl** | 45 – 60 | 43 |
| **[C_4_mim][BF_4_]** | 900 | 42 |
| **[C_4_mim][Tf_2_N]** | N/A | 44 |
